# Relationship between sarcopenia and cardiovascular disease among middle-aged and older adults with normal weight in China: functional limitation plays a mediating role

**DOI:** 10.1265/ehpm.24-00351

**Published:** 2025-06-04

**Authors:** Hui Cheng, Zhihui Jia, Jiaheng Chen, Yao Jie Xie, Jose Hernandez, Harry H.X. Wang

**Affiliations:** 1 School of Public Health, Sun Yat-Sen University, PR China; 2 School of Nursing, The Hong Kong Polytechnic University, Hong Kong SAR; 3 Joint Research Centre for Primary Health Care, The Hong Kong Polytechnic University, Hong Kong SAR; 4 Research Centre for Chinese Medicine Innovation, The Hong Kong Polytechnic University, Hong Kong SAR; 5 Green Templeton College, University of Oxford, Oxford, UK; 6 JC School of Public Health and Primary Care, Faculty of Medicine, The Chinese University of Hong Kong, Hong Kong SAR; 7 Usher Institute, College of Medicine and Veterinary Medicine, The University of Edinburgh, UK

**Keywords:** Sarcopenia, Cardiovascular disease, Functional limitation, Mediating effect, Middle-aged and older adults

## Abstract

**Background:**

Cardiovascular disease (CVD) is the predominant cause of mortality in China. However, the mechanisms linking sarcopenia to CVD remain poorly understood, particularly in normal-weight populations. Individuals with the absence of overweight or obesity may tend to experience missed opportunities for timely intervention. This study aimed to investigate the longitudinal association between sarcopenia and incidence of new-onset CVD in a normal-weight population, and to examine the mediating effect of functional limitation in this relationship.

**Methods:**

We conducted a closed-cohort analysis using a nationwide sample of 4,147 middle-aged and older adults with normal weight in China. We performed Cox proportional hazards regression analysis to explore the associations of baseline sarcopenia with incident CVD. The difference method was applied to estimate the mediation proportion of functional limitation in this association.

**Results:**

Over a mean follow-up period of 7.62 years, CVD occurred in 835 participants. In the multivariable-adjusted Cox model, individuals with sarcopenia exhibited a significantly higher likelihood of developing incident CVD compared to those without sarcopenia (adjusted hazard ratio [aHR] = 1.45, 95% confidence interval [CI]: 1.21–1.73, *P* < 0.001). Similar associations were observed for the incidence of heart disease and stroke. Functional limitation accounted for approximately 15.0% of the total effect of sarcopenia on incident CVD (*P* < 0.001).

**Conclusions:**

Sarcopenia exerts both direct and indirect effects on incident CVD among middle-aged and older adults who are normal weight, with functional limitation serving as a significant mediator. Interventions targeting both sarcopenia and functional limitation may offer a promising strategy for enhancing cardiovascular health in this population.

**Supplementary information:**

The online version contains supplementary material available at https://doi.org/10.1265/ehpm.24-00351.

## Graphical Abstract

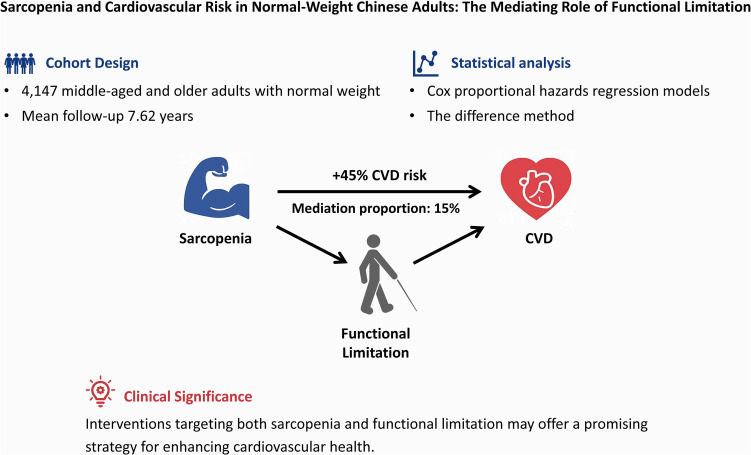

## Introduction

Cardiovascular diseases (CVD) are the leading cause of global mortality and major contributors to disability, representing a major public health problem. In China, CVD contributed to 43.5% of total deaths and 24.9% of total disability-adjusted life years in 2021 [1]. Notably, the number of CVD-related deaths has continued to increase, escalating from approximately 3.1 million in 2005 to 4.6 million in 2020, with substantial burden of premature mortality associated with CVD [2]. The challenge underscores the critical need to identify and address modifiable risk factors that may influence the development of CVD, thereby informing primary prevention strategies.

Sarcopenia, an age-related pathological condition characterised by the degenerative loss of skeletal muscle mass, strength, and physical function, has emerged as a significant public health concern in ageing societies. Sarcopenia is associated with frailty, increased fall risk, nutritional deficiencies, and greater mortality [3]. Previous studies have reported considerable variation in the prevalence of sarcopenia among Asian elderly populations, ranging from 6.8% to 25.7% [4–8], and in particular, from 34% to 66% among those with heart failure [9]. Although studies suggested a relationship between sarcopenia and CVD, evidence from large-scale longitudinal studies on the underlying mechanisms linking these conditions remain relatively scanty. Besides, existing research predominantly focused on the interplay between sarcopenia and obesity in the development of CVD. Individuals with the absence of overweight or obesity may therefore tend to experience missed opportunities for timely intervention to prevent a CVD event.

Functional limitation, which reflects a significant impairment in performing essential daily activities, is closely linked to reduced quality of life and increased morbidity and mortality [10, 11]. In China, nearly one in five middle-aged and older adults have functional limitation [12]. Given its quantifiable nature and clinical relevance to health outcomes, functional limitation has gained increasing attention. A growing body of evidence suggests that muscle strength serves as an important determinant of daily performance [13–15], with muscle preservation representing an effective strategy for mitigating functional decline. Studies also indicate that older adults with functional limitation face elevated CVD risk [10], suggesting that functional limitation may serve as a potential mediator in the sarcopenia-CVD pathway.

In the present study, we aimed to investigate the longitudinal association between baseline sarcopenia and incidence of new-onset CVD in a normal-weight population, while examining whether functional limitation mediates this relationship.

## Methods

### Study design and setting

We performed a retrospective cohort analysis using nationally representative data from the China Health and Retirement Longitudinal Study (CHARLS, 2011–2020), a comprehensive nationwide ageing survey designed after the US Health and Retirement Study and other internationally developed ageing surveys, while adapted for China’s health context. The baseline survey was commenced in 2011 (Wave 1). Subsequent surveys were repeatedly conducted in 2013 (Wave 2), 2015 (Wave 3), 2018 (Wave 4), and 2020 (Wave 5). Detailed descriptions of the sampling frame and household interview procedures were described elsewhere [16]. In brief, the study employed a multistage, region-stratified, probability proportional to size sampling across 28 out of a total of 31 provinces in China, encompassing 150 county-level districts, 450 neighbourhood- and village-level units, and over 10 thousand households. Individual data on socio-demographics, lifestyle, self-report of physician-diagnosed chronic conditions, activities of daily living (ADL), and instrumental activities of daily living (IADL) were collected via face-to-face interviews using computer-assisted personal interviewing technology, which enabled interviewers to directly input participants’ responses into a digital questionnaire system, obviating the need for paper-based data collection instruments. Health information on anthropometric and clinical parameters were also assessed through standardised tests of physical performance and assays of venous blood [16].

### Participants

**Fig. 1:**
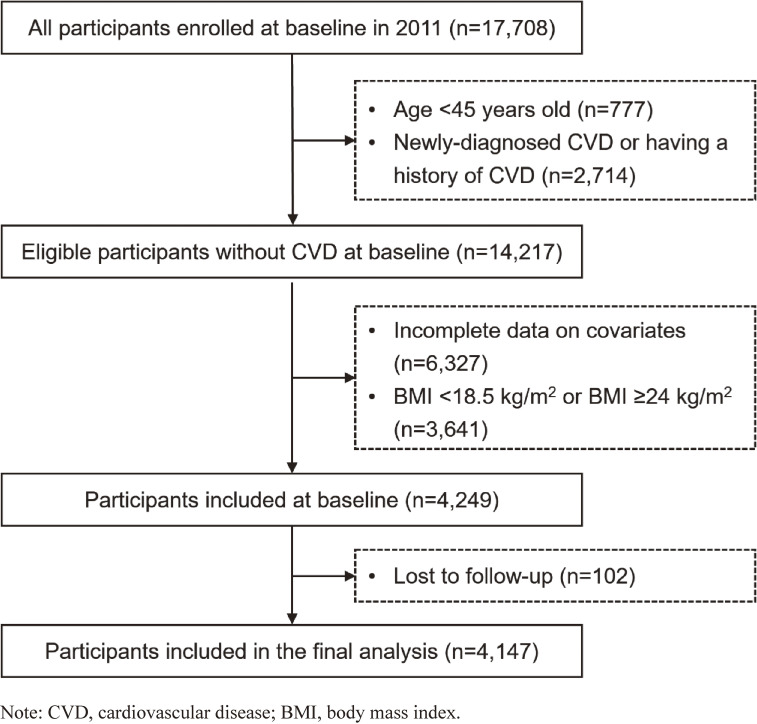
Diagram of study flow

A total of 17,708 participants were enrolled at baseline, of whom 16,931 were over 45 years of age. We excluded those with: (i) a clinical diagnosis of CVD (n = 2,714); (ii) incomplete data on covariates (n = 6,327); (iii) body mass index (BMI) <18.5 kg/m^2^ or ≥24 kg/m^2^ (n = 3,641); and (iv) loss to follow-up (n = 102). This yielded a total of 4,147 participants who met the eligibility criteria and were included in the closed-cohort analysis (Fig. 1).

### Measurements

Sarcopenia was diagnosed according to the 2019 Asian Working Group criteria, requiring low muscle mass in conjunction with decreased muscle strength or reduced physical performance [17]. Muscle strength was assessed via dominant-hand grip strength (Yuejian™ WL-1000 hydraulic handgrip dynamometer, Nantong, China) [16], with cut-offs of <28 kg for men and <18 kg for women [17]. Appendicular skeletal muscle mass (ASM) was calculated by a validated Chinese anthropometric equation: ASM = 0.193*weight (kg) + 0.107*height (cm) − 4.157*sex (1 for male; 2 for female) − 0.037*age (years) − 2.631 [18]. Previous studies have demonstrated strong concordance between ASM estimates derived from the equation and those obtained via dual-energy X-ray absorptiometry measurements [18, 19]. Low muscle mass was defined as sex-specific lowest 20% of the distribution of the ASM/height^2^ index [12], representing <7.00 kg/m^2^ for men and <5.15 kg/m^2^ for women in our study population. Anthropometrics were obtained using a portable stadiometer (Seca™ 213 stadiometer, Hangzhou, China) and a calibrated digital scale (Omron™ HN-286 scale, Yangzhou, China), with participants in light clothing and without shoes. Physical performance was examined through gait speed and chair stand test. Study participants were instructed to complete two 2.5 m walks at normal pace, with total time recorded. Participants were also guided to perform five chair rises at maximum speed, with completion time measured from initial standing to final seated position [16]. Low physical performance was defined as either a gait speed <1.0 m/s or a timed test of five chair rises ≥12 seconds [20]. Functional limitation was defined as self-reported difficulty in ≥1 ADL (dressing, eating, bathing or showering, getting in/out of bed, toileting, and controlling urination and defecation [21]) or ≥1 IADL (housekeeping, cooking, shopping, managing finances, and taking medication [22]).

### Outcomes and covariates

Primary outcomes were the first incidence of physician-diagnosed CVD (heart disease/stroke) identified through structured questions about cardiac (i.e., heart attack, coronary heart disease, angina, congestive heart failure, or other heart problems) and cerebrovascular events (i.e., stroke) during follow-up. Study participants enrolled at baseline were followed until they had newly diagnosed heart disease or stroke, or the recorded attendance at the most recent wave of follow-up, whichever came first.

Covariate data encompassed socio-demographics (age, sex, place of residence, and education level) and self-reported lifestyle (cigarette smoking and alcohol drinking) collected by computer-assisted personal interviewing, and routine clinical measurements (waist circumference and metabolic biomarkers) [16]. Central obesity was defined as a waist circumference exceeding 90 cm in males and 85 cm in females, according to Chinese population-specific diagnostic criteria [23]. Blood pressure (BP) was measured in a seated posture using a routinely validated automatic BP monitor (Omron™ HEM-7112/7200 Monitor), and the arm with the higher pressure was used. Three measurements were obtained at 45-second intervals, with the mean value recorded for analysis [16]. A venous blood sample at fasting was collected to measure metabolic biomarkers, including C-reactive protein, serum creatinine, and serum uric acid, following standard operating procedures [24].

### Statistical analysis

Data are presented as n (%) for categorical variables and as mean (SD) or median (IQR; 25^th^ to 75^th^ percentiles) for continuous variables where appropriate. Baseline characteristics were compared between sarcopenia and non-sarcopenia groups using the two-sample *t* test, the nonparametric Wilcoxon rank sum test, or the chi-square test, as appropriate. The cumulative hazard of new-onset CVD (including components) was determined by the Kaplan-Meier plot, and the two-sided logrank test was used for the comparison of curves between those with and without sarcopenia at baseline. Incidence rates of CVD events were reported per 1000 person-years. Cox proportional hazards regression models were constructed to estimate the risk of incident CVD after adjusting for demographic, socioeconomic, lifestyle, and health-related factors. We tested the proportional hazards assumption for model fit using the scaled Schoenfeld residuals. Stratified analyses were performed to examine the potential effect modification by age (<65 vs. ≥65 years), sex (male vs. female), place of residence (urban vs. rural), cigarette smoking (current smoker vs. non-smoker), alcohol drinking (regular drinker vs. non-drinker), and the presence of baseline comorbidities including hypertension, diabetes, and dyslipidaemia. The interaction between sarcopenia and each stratifying variable was explored by inserting a two-factor interaction term into the regression model.

We employed the difference method to estimate the mediation proportion of functional limitation at Wave 1 in the relationship between sarcopenia and incident CVD (including components) by comparing fully-adjusted model estimates with and without the inclusion of the hypothesised mediator [25]. In the sensitivity analyses, we excluded incident CVD cases by the first follow-up visit were excluded to account for the possible reverse causality bias, and further excluded participants with central obesity given the role of abnormal adipose tissue distribution as a risk factor for CVD. Analyses were conducted using SAS (version 9.4, SAS Institute Inc, NC, USA) for data management and statistical analysis, and R (version 4.0.2, Core Team, Vienna, Austria) for graphical visualisation, with *P* < 0.05 considered statistically significant.

## Results

### Baseline characteristics of participants

Participants ranged in age from 45 to 95 years, with a mean age of 59.19 ± 9.29 years. CVD occurred in 835 participants (i.e., 377 males and 458 females) over a mean of 7.62 years of follow-up. Of 4,147 middle-aged and older adults with normal weight, the prevalence of sarcopenia was 16.52% (685/4,147). Compared to non-sarcopenic participants, those with sarcopenic at baseline were older (69.12 ± 8.19 vs. 57.23 ± 8.17 years), more rural-residing (76.35% vs. 68.92%), and had lower education levels (88.91% vs. 69.41%). They also exhibited higher hypertension prevalence (39.71% vs. 30.44%), greater functional limitation (42.34% vs. 28.28%), and elevated biomarker levels, including systolic blood pressure (132.30 ± 23.80 vs. 126.70 ± 20.07 mmHg) and C-reactive protein [2.93 (IQR 2.25–3.61) vs. 2.55 (2.26–2.83) mg/dL], compared to their counterparts without sarcopenic at baseline (all *P* < 0.05; Table 1).

**Table 1:** Characteristics of participants by sarcopenia at baseline

**Characteristics**	**Sarcopenia** **(n = 685)**	**Non-sarcopenia** **(n = 3462)**	***P* value**
**Age, years**	69.12 ± 8.19	57.23 ± 8.17	<0.001
**Sex**			0.660
Male	356 (51.97)	1831 (52.89)	
Female	329 (48.03)	1631 (47.11)	
**Place of residence**			<0.001
Urban	162 (23.65)	1076 (31.08)	
Rural	523 (76.35)	2386 (68.92)	
**Education level**			<0.001
Elementary school or below	609 (88.91)	2403 (69.41)	
Middle school or above	76 (11.09)	1059 (30.59)	
**Cigarette smoking,**			0.813
Current smoker	249 (36.35)	1275 (36.83)	
Non-smoker	436 (63.65)	2187 (63.17)	
**Alcohol drinking**			0.065
Regular drinker	235 (34.31)	1317 (38.04)	
Non-drinker	450 (65.69)	2145 (61.96)	
**Comorbidities**			
Hypertension	272 (39.71)	1054 (30.44)	<0.001
Diabetes	83 (12.12)	460 (13.29)	0.407
Dyslipidaemia	190 (27.74)	1177 (34.00)	0.001
**Functional limitation**	290 (42.34)	979 (28.28)	<0.001
**BMI, kg/m^2^**	19.86 ± 1.02	21.84 ± 1.35	<0.001
**WC, cm**	76.91 ± 8.48	80.23 ± 9.53	<0.001
**SBP, mmHg**	132.20 ± 23.80	126.70 ± 20.07	<0.001
**DBP, mmHg**	72.42 ± 11.93	74.02 ± 11.61	0.001
**FPG, mg/dL**	107.20 (104.90, 109.60)	107.40 (106.30, 108.60)	0.368
**CRP, mg/dL**	2.93 (2.25, 3.61)	2.55 (2.26, 2.83)	0.010
**Scr, mg/dL**	0.78 (0.77, 0.80)	0.78 (0.77, 0.79)	0.542
**SUA, mg/dL**	4.40 ± 1.31	4.38 ± 1.22	0.769

Note: BMI, body mass index; WC, waist circumference; SBP, systolic blood pressure; DBP, diastolic blood pressure; FPG, fasting plasma glucose; CRP, C-reactive protein; Scr, serum creatinine; SUA, serum uric acid.

Data are presented as n (%) for categorical variables and as mean ± standard deviation or median (interquartile range [IQR; 25^th^ to 75^th^ percentiles]) for continuous variables as appropriate. The two-sample *t* test, the nonparametric Wilcoxon rank sum test, or the chi-square test was used for between-group comparisons of participants with and without sarcopenia at baseline, where appropriate.

### Associations between baseline sarcopenia and first incident CVD

The incidence of new-onset CVD was reached at a rate of 26.42 per 1,000 person-years of follow-up in overall participants (i.e., 35.96 and 24.79 per 1,000 person-years for those with and without sarcopenia at baseline, respectively; Table 2). Participants with baseline sarcopenia exhibited higher incidence rates of both heart disease (20.0% vs. 14.9%) and stroke (7.3% vs. 6.1%) compared to their counterparts who were free of sarcopenia. The highest cumulative hazard of CVD, including heart disease and stroke, was observed at 9 years in participants with sarcopenia at baseline when compared to that in non-sarcopenic participants (Logrank test, all *P* < 0.05; Supplementary Fig. S1). In the fully adjusted model, sarcopenia predicted a 45% greater risk of new-onset CVD (adjusted hazard ratio [aHR] = 1.45, 95% CI: 1.21–1.73, *P* < 0.001), with similar associations for incident heart disease (aHR = 1.52, 95% CI: 1.24–1.85, *P* < 0.001) and stroke (aHR = 1.39, 95% CI: 1.01–1.93, *P* = 0.046). These findings persisted after excluding individuals who developed CVD by the first follow-up visit and those with central obesity at baseline (Supplementary Tables S1–S2).

**Table 2:** Incidence rate of CVD at follow-up and association between baseline sarcopenia and new-onset CVD

**Variables**	**Cases, n**	**Incidence rate^a^**	**Model 1**		**Model 2**		**Model 3**	
		
			**HR (95%CI)**	***P* value**	**aHR (95%CI)**	***P* value**	**aHR (95%CI)**	***P* value**
**CVD at follow-up**								
Non-sarcopenia at baseline	668	24.79	1.00 (reference)		1.00 (reference)		1.00 (reference)	
Sarcopenia at baseline	167	35.96	1.47 (1.24, 1.74)	<0.001	1.41 (1.18, 1.68)	<0.001	1.45 (1.21, 1.73)	<0.001
**Heart disease at follow-up**								
Non-sarcopenia at baseline	516	18.72	1.00 (reference)		1.00 (reference)		1.00 (reference)	
Sarcopenia at baseline	137	28.39	1.54 (1.28, 1.86)	<0.001	1.50 (1.23, 1.82)	<0.001	1.52 (1.24, 1.85)	<0.001
**Stroke at follow-up**								
Non-sarcopenia at baseline	211	7.39	1.00 (reference)		1.00 (reference)		1.00 (reference)	
Sarcopenia at baseline	50	9.83	1.38 (1.01, 1.87)	0.043	1.31 (0.95, 1.80)	0.095	1.39 (1.01, 1.93)	0.046

Note: CVD, cardiovascular disease; aHR, adjusted hazard ratio.

The full cohort included 3,462 non-sarcopenic individuals and 685 sarcopenic individuals at baseline.

Model 1 referred to Cox proportional hazards models with no adjustment. Model 2 was adjusted for age, sex, place of residence, education level, cigarette smoking, and alcohol drinking. Model 2 was further adjusted for waist circumference, C-reactive protein, serum creatinine, serum uric acid, and the presence of comorbidities including hypertension, diabetes, and dyslipidaemia.

^a^per 1000 person-years.

### Subgroup analysis

**Fig. 2:**
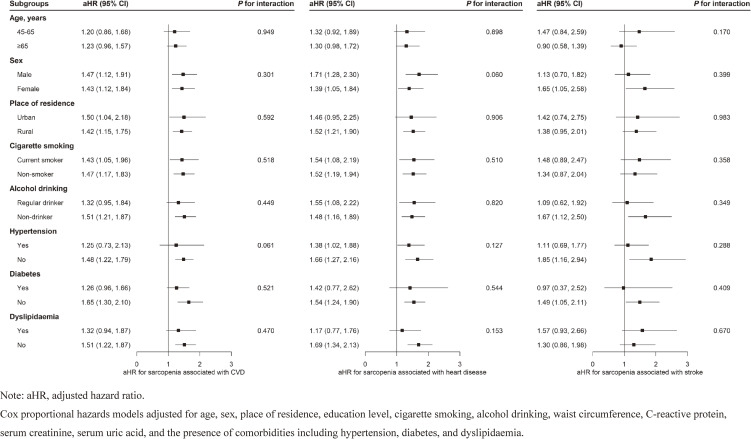
Association between sarcopenia and incident CVD across population subgroups

When participants were classified by age, sex, place of residence, cigarette smoking, alcohol drinking, and the presence of hypertension, diabetes and dyslipidaemia, the associations of baseline sarcopenia and incident CVD (including components) observed in the main analysis remained consistent across all subgroups. Multivariable-adjusted Cox model showed no significant effect modification by these stratifying variables for either composite CVD or its components (all *P* for interaction >0.05; Fig. 2).

### Mediating role of functional limitation

**Fig. 3:**
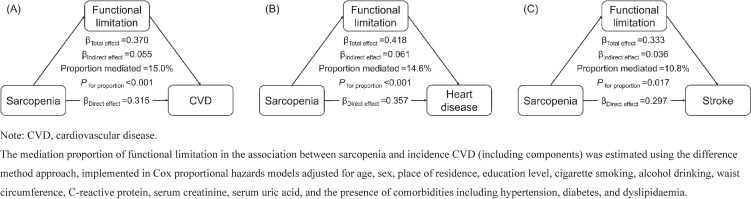
Mediation model of the relationship among sarcopenia, functional limitation, and incident CVD

The mediation analyses revealed that functional limitation significantly mediated the relationship between sarcopenia and incident CVD, accounting for approximately 15.0% of the association in the fully adjusted model. Further analysis of the association between sarcopenia and individual components of CVD demonstrated that functional limitation explained over 10% of the increased risks of both new-onset heart disease and stroke (all *P* for proportion <0.05; Fig. 3).

## Discussion

### Main findings

In this 9-year longitudinal cohort study of middle-aged and older adults in China, our study revealed significant associations between baseline sarcopenia and incidence of new-onset CVD. Functional limitation was found to partially mediate this relationship. Our findings highlight sarcopenia as a key cardiovascular health risk factor, even in non-obese individuals, implying that early interventions targeting muscle preservation and functional capacity may mitigate CVD risk.

### Relationship with other studies

As common age-related conditions, sarcopenia and CVD pose increasing challenges in public health practice and research. Previous studies support a positive association between sarcopenia and CVD [12, 26–28]. A recent meta-analysis of cross-sectional studies in Asian populations documented elevated CVD risk in sarcopenic individuals [26]. Our results align with a previous longitudinal study [12], while extending the evidence with significantly longer follow-up (9 vs. 3.6 years) and sensitivity analyses excluding early CVD cases to strengthen causal inference. Beyond Asia, reports from other global regions corroborated the relationship between sarcopenia and CVD [27, 28]. Multi-ethnic population data from the US National Health and Nutrition Examination Survey linked sarcopenia to CVD [27], as well as to poorer cardiovascular health markers, including smoking, physical activity, dietary intake, BMI, lipid profile, blood pressure, and fasting glucose level [28]. Similarly, cohort studies from the UK Biobank reported that sarcopenia increased the risk of developing CVD in patients with diabetes [29], and that low grip strength (as part of the diagnostic criterion for sarcopenia) linked to incident heart failure [30]. Collectively, findings across different populations underscored the ubiquitous role of sarcopenia in cardiovascular pathophysiology.

Previous findings also suggested that sarcopenia was independently predictive of new-onset CVD and cardiovascular mortality in people with comorbidities such as diabetes and chronic kidney disease [29, 31, 32]. However, this association was not statistically significant in participants with baseline diabetes or dyslipidaemia in our study, likely due to reduced statistical power from stratified sample sizes. Significant associations between sarcopenia and incident CVD were observed in the absence of diabetes and dyslipidaemia at baseline, with interaction tests showing no significant between-group differences. From a population perspective, our findings emphasise that muscle loss should not be neglected in middle-aged and older adults, even with the absence of chronic comorbidities.

Existing research has been dominated by a focus on the interplay between sarcopenia and obesity in CVD pathogenesis. Previous studies manifested that sarcopenia exacerbated CVD risk in obese population [9, 33, 34]. The loss of muscle strength and mass may lead to increased adiposity, insulin resistance, and chronic inflammation, thereby predisposing individuals to impaired cardiovascular health [9]. Notably, in our analysis, we identified sarcopenia as a significant risk factor for CVD even in healthy-weight individuals–a demographic group frequently neglected in clinical interventions. After adjusting for obesity, we observed a partial mediating effect of functional limitation, suggesting that sarcopenia may impact cardiovascular health in part through physical dysfunction. A meta-analysis of prospective studies reported associations of muscle mass, muscle strength, and physical performance with functional capacity including ADL and IADL [35], while a substantial body of research has linked functional limitation to cardiovascular events and mortality [10, 36, 37], thereby corroborating the findings on the mediating role of functional limitation observed in our study population.

The underlying mechanisms are multifaceted. Sarcopenia-induced functional decline may result in reduced physical activity [13], poor sleep efficiency or sleep irregularity [38], and thereafter tended to increase CVD and mortality risks [39]. From a social-psychological perspective, functional limitation in later life is associated with a loss of autonomy and an increased dependence on carers, thus predisposing individuals to lower engagement in social activities and fostering social isolation [40, 41]. Those experiencing physical functional limitation are at a heightened risk of developing depressive symptoms, anxiety, and suicidal ideation [42, 43]. Such psychological distresses may, in turn, trigger pathways that involve inflammatory cascades, platelet activation, thrombosis, and autonomic dysfunction, which may all influence risks for stroke and myocardial ischaemia [44–46]. It is noteworthy that the higher heart disease incidence (vs. stroke) seen in our study likely reflects our broad case definition, encompassing heart attack, coronary heart disease, angina, congestive heart failure, or other cardiac conditions. Variations across studies in the incidence rate of heart disease may explained by differences in population characteristics and study criteria. Nevertheless, our findings align with a previous study based on CHALRS data reporting comparable rates in the incidence of heart disease and stroke [34].

### Implications for research and clinical practice

Our findings demonstrate a significant association between sarcopenia and incidence of new-onset CVD, which underscores the importance of sarcopenia screening in community health assessments and routine clinical practice. Since sarcopenia is modifiable through intervention [9], identification of at-risk individuals is necessary for a tailored and timely approach to muscle preservation, thereby potentially reducing cardiovascular events. The mediating role of functional limitation suggests that targeted interventions aimed at improving physical function may benefit cardiovascular health. Evidence-based exercise prescriptions such as aerobic and resistance training can enhance muscle strength and reduce the risk of functional disabilities [9, 47], which may require nutritional strategies to optimise protein intake and improve dietary quality for supporting muscle and cardiovascular health [9]. Raising awareness among middle-aged and older adults regarding the importance of maintaining muscle health and functional independence–through targeted exercise and dietary measures–can contribute to improved cardiovascular outcomes and healthy ageing.

### Strengths and weaknesses of the study

Our study carries a clinically meaningful message regarding the association of sarcopenia with cardiovascular health in normal-weight middle-aged and older population. The study also represents a pioneering investigation into the mediating role of functional limitation in this association, offering insights for cardiovascular prevention. Data were drawn from a rigorously designed and conducted nationwide survey specifically for middle-aged and older adults, with the outcomes of interest examined over a considerable follow-up period. This study has some limitations which merit consideration. First, while longitudinal mediation analysis would be ideal considering the temporal sequence of causality, unavailable serial data on functional limitation at each follow-up visit may preclude causal inference across timepoints. Second, self-reported functional measures may introduce recall bias, while the unassessed severity and duration aspects warrant further investigation. Third, subtypes of heart conditions were not differentiated due to methodological constraints in the original questionnaire design, which precluded our ability to perform subtype-level analysis of sarcopenia-cardiac disease relationships. Future research is warranted to differentiate pathological categories (e.g., congestive heart failure vs. coronary artery disease) to help elucidate the distinct mechanistic pathways and enable more precisely targeted prevention strategies. Fourth, unmeasured confounders such as physical activities, dietary regimes, environmental exposures, and healthcare utilisation may exert cohort effects. Last but not least, culture and lifestyle specificities may impact the generalisability of our findings to other populations, thereby requiring further validation in diverse multi-ethnic cohorts.

## Conclusion

In conclusion, our study demonstrates the association between sarcopenia and incidence of new-onset CVD in a normal-weight population, while revealing the significant mediating role of functional limitation. These pioneering findings emphasise the importance of considering muscle health in cardiovascular risk assessments. Interventions targeting both sarcopenia and functional limitation may offer a promising strategy for enhancing cardiovascular health in middle-aged and older adult population.

## Abbreviations

ADL: Activities of Daily Living
ASM: Appendicular Skeletal Muscle Mass
AWGS: Asian Working Group for Sarcopenia
BP: Blood pressure
BMI: Body Mass Index
CHARLS: China Health and Retirement Longitudinal Study
CVD: Cardiovascular Disease
IADL: Instrumental Activities of Daily Living

## Supplementary information

**Additional file 1:** **Supplementary Figure S1** Cumulative hazard of new-onset CVD by baseline sarcopenia.

**Additional file 2:** **Supplementary Table S1** Sensitivity analysis by excluding incident CVD cases by the first follow-up visit. **Supplementary Table S2** Sensitivity analysis by excluding participants with central obesity at baseline.

## Declarations

### Availability of data and materials

The datasets used and/or analysed during the current study are available from the corresponding author upon reasonable request.
