# Supplementary material for: Relationship between sarcopenia and cardiovascular disease among middle-aged and older adults with normal weight in China: functional limitation plays a mediating role
Source: Environ Health Prev Med. 2025 Jun 4;30:46. doi: 10.1265/ehpm.24-00351 (PMC12170099; doi:10.1265/ehpm.24-00351)
Supplement: Supplementary file 1 — Additional file 1: Supplementary Figure S1 Cumulative hazard of new-onset CVD by baseline sarcopenia. [file ehpm-30-046-s001.docx]

***Supplementary Material***

**Relationship between sarcopenia and cardiovascular disease among middle-aged and older adults with normal weight in China: Functional limitation plays a mediating role**

**Supplementary Figure S1** Cumulative hazard of new-onset CVD by baseline sarcopenia

**Supplementary Figure S1 Cumulative hazard of new-onset CVD by baseline sarcopenia**


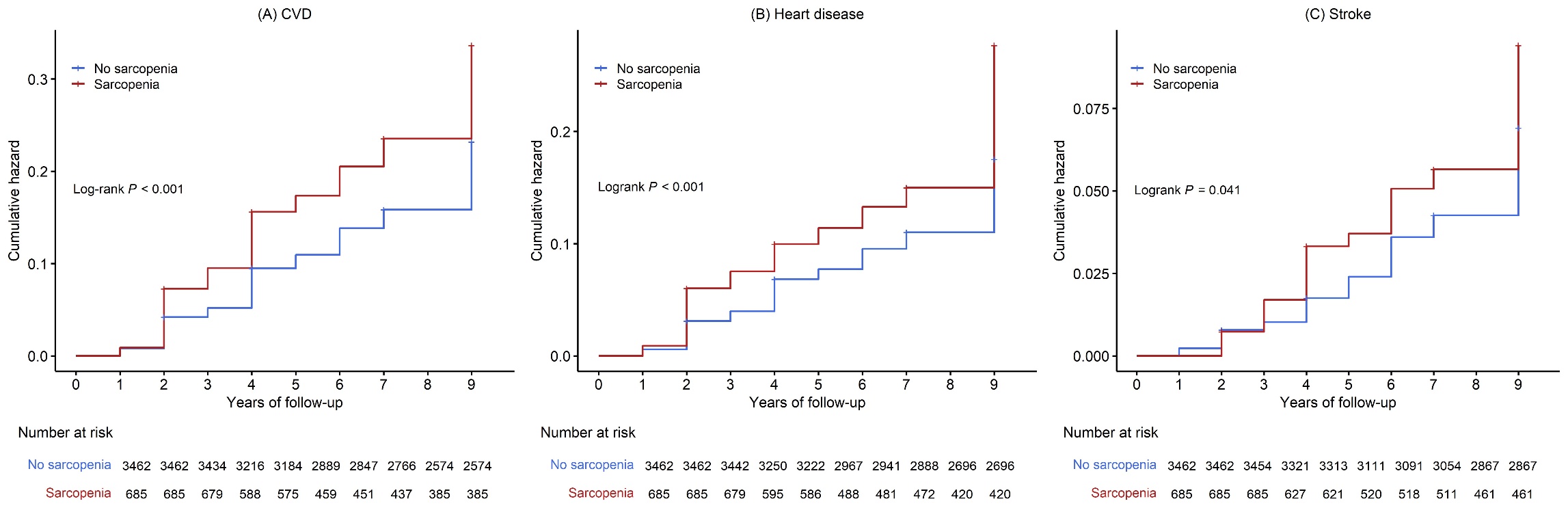


Note: CVD, cardiovascular disease.
