# Supplementary material for: Relationship between sarcopenia and cardiovascular disease among middle-aged and older adults with normal weight in China: functional limitation plays a mediating role
Source: Environ Health Prev Med. 2025 Jun 4;30:46. doi: 10.1265/ehpm.24-00351 (PMC12170099; doi:10.1265/ehpm.24-00351)
Supplement: Supplementary file 2 — Additional file 2: Supplementary Table S1 Sensitivity analysis by excluding incident CVD cases by the first follow-up visit. Supplementary Table S2 Sensitivity analysis by excluding participants with central obesity at baseline. [file ehpm-30-046-s002.docx]

**Relationship between sarcopenia and cardiovascular disease among middle-aged and older adults with normal weight in China: Functional limitation plays a mediating role**

*Environmental Health and Preventive Medicine*

<https://doi.org/10.1265/ehpm.24-00351>

**Supplementary Table S1** Sensitivity analysis by excluding incident CVD cases by the first follow-up visit

**Supplementary Table S2** Sensitivity analysis by excluding participants with central obesity at baseline

**Supplementary Table S1 Sensitivity analysis by excluding incident CVD cases by the first follow-up visit**

| **Variables** | **Cases, n** | **Incidence rate^a^** | **Model 1** |  |  | **Model 2** |  |  | **Model 3** |  |
| --- | --- | --- | --- | --- | --- | --- | --- | --- | --- | --- |
|  |  |  | **HR (95%CI)** | ***P* value** |  | **aHR (95%CI)** | ***P* value** |  | **aHR (95%CI)** | ***P* value** |
| **CVD at follow-up** |  |  |  |  |  |  |  |  |  |  |
| Non-sarcopenia at baseline | 526 | 19.71 | 1.00 (reference) |  |  | 1.00 (reference) |  |  | 1.00 (reference) |  |
| Sarcopenia at baseline | 119 | 26.13 | 1.39 (1.14, 1.70) | 0.001 |  | 1.33 (1.08, 1.63) | 0.008 |  | 1.36 (1.10, 1.68) | 0.005 |
| **Heart disease at follow-up** |  |  |  |  |  |  |  |  |  |  |
| Non-sarcopenia at baseline | 396 | 14.62 | 1.00 (reference) |  |  | 1.00 (reference) |  |  | 1.00 (reference) |  |
| Sarcopenia at baseline | 92 | 19.64 | 1.41 (1.13, 1.77) | 0.003 |  | 1.34 (1.06, 1.70) | 0.015 |  | 1.36 (1.07, 1.73) | 0.013 |
| **Stroke at follow-up** |  |  |  |  |  |  |  |  |  |  |
| Non-sarcopenia at baseline | 171 | 6.20 | 1.00 (reference) |  |  | 1.00 (reference) |  |  | 1.00 (reference) |  |
| Sarcopenia at baseline | 37 | 7.80 | 1.33 (0.93, 1.90) | 0.116 |  | 1.29 (0.90, 1.85) | 0.168 |  | 1.42 (0.98, 2.06) | 0.068 |

Note: CVD, cardiovascular disease; aHR, adjusted hazard ratio.

The sensitivity cohort included 3,320 non-sarcopenic individuals and 637 sarcopenic individuals at baseline, after excluding participants who had new-onset CVD by the first follow-up visit (n=190).

Model 1 referred to Cox proportional hazards models with no adjustment. Model 2 was adjusted for age, sex, place of residence, education level, cigarette smoking, and alcohol drinking. Model 2 was further adjusted for waist circumference, C-reactive protein, serum creatinine, serum uric acid, and the presence of comorbidities including hypertension, diabetes, and dyslipidaemia.

^a^ per 1000 person-years.

**Supplementary Table S2 Sensitivity analysis by excluding participants with central obesity at baseline**

| **Variables** | **Cases, n** | **Incidence rate^a^** | **Model 1** |  |  | **Model 2** |  |  | **Model 3** |  |
| --- | --- | --- | --- | --- | --- | --- | --- | --- | --- | --- |
|  |  |  | **HR (95%CI)** | ***P* value** |  | **aHR (95%CI)** | ***P* value** |  | **aHR (95%CI)** | ***P* value** |
| **CVD at follow-up** |  |  |  |  |  |  |  |  |  |  |
| Non-sarcopenia at baseline | 408 | 18.33 | 1.00 (reference) |  |  | 1.00 (reference) |  |  | 1.00 (reference) |  |
| Sarcopenia at baseline | 103 | 24.76 | 1.42 (1.14, 1.76) | 0.002 |  | 1.34 (1.07, 1.68) | 0.010 |  | 1.34 (1.07, 1.69) | 0.011 |
| **Heart disease at follow-up** |  |  |  |  |  |  |  |  |  |  |
| Non-sarcopenia at baseline | 308 | 13.65 | 1.00 (reference) |  |  | 1.00 (reference) |  |  | 1.00 (reference) |  |
| Sarcopenia at baseline | 84 | 19.71 | 1.52 (1.20, 1.94) | 0.001 |  | 1.45 (1.13, 1.87) | 0.004 |  | 1.45 (1.12, 1.87) | 0.005 |
| **Stroke at follow-up** |  |  |  |  |  |  |  |  |  |  |
| Non-sarcopenia at baseline | 134 | 5.85 | 1.00 (reference) |  |  | 1.00 (reference) |  |  | 1.00 (reference) |  |
| Sarcopenia at baseline | 28 | 6.47 | 1.16 (0.78, 1.75) | 0.464 |  | 1.13 (0.75, 1.71) | 0.569 |  | 1.20 (0.78, 1.83) | 0.405 |

Note: CVD, cardiovascular disease; aHR, adjusted hazard ratio.

The sensitivity cohort included 2,744 non-sarcopenic individuals and 577 sarcopenic individuals at baseline, after excluding participants who had new-onset CVD by the first follow-up visit (n=190) and participants who had central obesity at baseline (n=636).

Model 1 referred to Cox proportional hazards models with no adjustment. Model 2 was adjusted for age, sex, place of residence, education level, cigarette smoking, and alcohol drinking. Model 2 was further adjusted for waist circumference, C-reactive protein, serum creatinine, serum uric acid, and the presence of comorbidities including hypertension, diabetes, and dyslipidaemia.

^a^ per 1000 person-years.
